# Comparing a Clinician Assisted and App-Supported Positive Psychiatry Behavioral Activation Intervention

**DOI:** 10.1093/geroni/igaa057.1203

**Published:** 2020-12-16

**Authors:** Ariane Massie, Keri-Leigh Cassidy, Michael Vallis, David Conn, Daria Parsons, Julie Spence Mitchell, Claire Checkland, Kiran Rabheru

**Affiliations:** 1 York University, North York, Ontario, Canada; 2 Dalhousie University, Dalhousie University, Nova Scotia, Canada; 3 Dalhousie University, Halifax, Nova Scotia, Canada; 4 University of Toronto, Toronto, Ontario, Canada; 5 Canadian Coalition for Seniors Mental Health, Markham, Ontario, Canada; 6 University of Ottawa, Ottawa, Ontario, Canada

## Abstract

Positive psychiatry offers a unique approach to promote brain health and well-being in aging populations. Health interventions are increasingly becoming available using self-guided apps, however, little is known about the effectiveness of app technology or the difference between in-person versus self-guided app methodology for behavioural activation. The objective of this study was to investigate the difference in users and outcomes between two formats of a positive psychiatry intervention to promote brain health and well-being in later-life: (1) clinician-assisted, and (2) independent app use for self-management. As part of a larger national knowledge translation intervention two methods of a behavioural activation intervention (Clinician-assisted vs. Independent app use) were retrospectively compared. Main outcomes were patient characteristics (age, sex, and completion rate), psychological outcomes (health and resilience, and well-being), and behavioural outcomes (goal attainment, and items of goal SMART-ness). Clinician-assisted patients (n=254) were more likely to be male, older, and had lower health and resilience scores at baseline than Independent app users (n=333). Clinician-assisted patients had notably higher completion rates (99.2% vs. 10.8%). Psychological outcomes were similar regardless of intervention method for those who completed the intervention. Clinician-assisted patients had higher rates of goal attainment and goal SMART-ness. A preliminary goal setting methodology for effective behavioural activation, to promote brain health and wellness, is given. Clinician-patient relationships were found to be an important factor for intervention completion, caution is given for app use referral. Results indicate a need for further exploration to determine best practices for health app use in clinical practice.

